# Supplementary material for: Face mask use in the general population and optimal resource allocation during the COVID-19 pandemic
Source: Nat Commun. 2020 Aug 13;11:4049. doi: 10.1038/s41467-020-17922-x (PMC7426871; doi:10.1038/s41467-020-17922-x)
Supplement: Supplementary file 1 — Supplementary Information [file 41467_2020_17922_MOESM1_ESM.pdf]

Supplementary Information for “*Face mask use in the general population and optimal resource allocation during the COVID-19 pandemic*”

Worby & Chang

Supplementary Methods
Supplementary Table 1
Supplementary Figures 1-11
Supplementary References

Supplementary Methods

We consider a population of size *N*, in which each individual has a status as a mask-wearer or a non wearer. For each status *f* (*f*=*m* for mask wearer, *f*=*n* for non wearer), let S^f, E^f, be the number of susceptible and exposed (but not infectious) individuals. Let I_P^f, I_A^f and I_S^f be the number of pre-symptomatic, asymptomatic/mildly symptomatic and fully symptomatic individuals respectively. Finally, let *R* and *D* be the number of recoveries and deaths. Fully symptomatic cases are associated with an infection rate of β_S, while pre-symptomatic and mildly/asymptomatic infections have a lower infection rate β_A; we assume $\beta_S = 2\beta_A$. Progression from exposed to pre-symptomatic occurs at rate α_1, and progression to either I_S or I_A occurs at rate α_2. The average incubation period ($1/\alpha_1$+$1/\alpha_2$) is assumed to be 6 days [1-3]. The probability of developing only mild symptoms or no symptoms among age group *x* is P_a. Removal occurs at rate γ_S and γ_A respectively. The proportion of fatalities among fully symptomatic cases in age group *x* is assumed to be p_D. We assumed an R_0 of 2.5 across models[4,5]. In order to calculate the infection rates in our models with multiple infected compartments, we derived the dominant eigenvalue of the next generation matrix [6] in terms of the model parameters:

$$R_0 = \frac{\beta_A}{\alpha_2} + \frac{\beta_A P_a}{\gamma_A} + \frac{\beta_S(1 - P_a)}{\gamma_S} \qquad (1)$$

so that if $\gamma_S = \gamma_A = \gamma$, and $\beta_A = \frac{\beta_S}{2}$:

$$\beta_S = R_0 \frac{2\alpha_2\gamma}{2\alpha_2 + \gamma - P_a\alpha_2} . \qquad (2)$$

Given that estimates for the proportion of asymptomatic infections vary considerably, including 18% based on data from the Diamond Princess cruise ship (with a high proportion of elderly people) [28], 25% according to the director of the US CDC [29], 30% based on Japanese evacuees from Wuhan [30], and even up to 78% based on limited reporting from China [31], we allowed this parameter to vary over a plausible range. We assume that mild/asymptomatic infections have an infection rate 50% lower than fully symptomatic infections.

Resource allocation model

We partition the population into age groups, comprising N_y and N_o young (<70 years old) and elderly (70+ years old) persons respectively. For each age group *x* (*x*=*y* for young, *x*=*o* for elderly), and status *f* (*f*=*m* for mask wearer, *f*=*n* for non-wearer), let S^{xf}, E^{xf}, be the number of susceptible and exposed (but not infectious) individuals, and we apply similar superscript notation for all other compartments. The probability of developing only mild symptoms or no symptoms among age group *x* is P_{ax}. The proportion of fatalities among fully symptomatic cases in age group *x* is assumed to be p_{xD}. Dynamics are governed by the following system of differential equations:

$$\frac{dS^{xn}}{dt} = -\frac{S^{xn}}{N}(\beta_A(I_P^{\cdot n} + I_A^{\cdot n} + r_t(I_P^{m} + I_A^{\cdot m})) + \beta_S(I_S^{\cdot n} + r_t I_S^{\cdot m})) \tag{3}$$

$$\frac{dS^{xm}}{dt} = -\frac{S^{xm}}{N} r_S(\beta_A(I_P^{\cdot n} + I_A^{\cdot n} + r_t(I_P^{\cdot m} + I_A^{\cdot m})) + \beta_S(I_S^{\cdot n} + r_t I_S^{\cdot m})) \tag{4}$$

$$\frac{dE^{xn}}{dt} = \frac{S^{xn}}{N}(\beta_A(I_P^{n} + I_A^{n} + r_t(I_P^{\cdot m} + I_A^{\cdot m})) + \beta_S(I_S^{\cdot n} + r_t I_S^{\cdot m})) - \alpha_1 E^{xn} \tag{5}$$

$$\frac{dE^{xm}}{dt} = \frac{S^{xm}}{N} r_S(\beta_A(I_P^{n} + I_A^{n} + r_t(I_P^{\cdot m} + I_A^{\cdot m})) + \beta_S(I_S^{\cdot n} + r_t I_S^{\cdot m})) - \alpha_1 E^{xm} \tag{6}$$

$$\frac{dI_P^{xs}}{dt} = \alpha_1 E^{xs} - \alpha_2 I_P^{xs} \tag{7}$$

$$\frac{dI_A^{xn}}{dt} = P_{ax}\alpha_2(1 - \delta m^*) I_P^{xn} - \gamma_A I_A^{xn} \tag{8}$$

$$\frac{dI_A^{xm}}{dt} = P_{ax}\alpha_2(\delta m^* I_P^{xn} + I_P^{xm}) - \gamma_A I_A^{xm} \tag{9}$$

$$\frac{dI_S^{xn}}{dt} = (1 - P_{ax})\alpha_2(1 - \delta m^*) I_P^{xn} - \gamma_S I_S^{xn} - \gamma_{xD} I_S^{xn} \tag{10}$$

$$\frac{dI_S^{xm}}{dt} = (1 - P_{ax})\alpha_2(\delta m^* I_P^{xn} + I_P^{xm}) - \gamma_S I_S^{xm} - \gamma_{xD} I_S^{xm} \tag{11}$$

$$\frac{dR^x}{dt} = (1 - \phi_x)\gamma_S I_S^{x\cdot} + \gamma_A I_A^{x\cdot} \tag{12}$$

$$\frac{dD^x}{dt} = \phi_x \gamma_S I_S^{x\cdot} \tag{13}$$

where m^* is an indicator function equal to 1 when there is a remaining supply of masks M, δ is the probability of detecting mild/asymptomatic infections, and ϕ_x is the probability of death for a symptomatic individual in age group x. As masks are provided to newly diagnosed cases, supplies are depleted at the following rate:

$$\frac{dM}{dt} = -\alpha_2(P_A \delta m^* I_P^{xn} + (1 - P_A) I_P^{xm}) \tag{14}$$

Mask supplies monotonically decline until zero is reached, at which point, no further masks are provided for detected cases. Models are run for three years; total cases and infections are counted until that point. While in some edge cases, the epidemic peak can be delayed until after this point, it is reasonable to assume that vaccination deployment will contribute to developing herd immunity by then, effectively ending the epidemic.

Supply & demand model

In this model (Supplementary Figure 11), we allow for movement between mask-wearing and non-mask-wearing status, depending on availability and demand, and masks must be continually acquired to remain a mask wearer. New masks are produced at a fixed rate B and mask supply decreases as people become mask wearers. We assume that the mask is worn on average for μ days before requiring replacement, and the rate of non-wearers acquiring masks depends on both demand (ω_A for healthy and asymptomatic individuals, or ω_S for symptomatic individuals) and current supply (M/N, the proportion of mask in the overall population). We assume that demand for masks increases with the number of reported cases in the population

up to a certain plateau, and as such modeled the relationship between ω_A and the number of symptomatic infections in the following sigmoidal function:

$$\omega_A = \frac{1}{1+e^{-k_1(I_S - k_2)}}. \tag{15}$$

The range of ω_A is [0, 1], k_1 represents the rate of demand increase, and k_2 represents the timing of demand, defined as the number of reported cases when half the population seeks face masks (i.e. higher k_2 means mask demand increases later in the outbreak). We allow ω_S to differ from ω_A, in order to explore the effects of recommending face mask use to the general population (ω_S=ω_A), or specifically to symptomatic individuals (ω_S>ω_A). This parameterization allows us to explore different demand dynamics, for example, panic buying (high k_1), delayed response to epidemic threat (high k_2), limited interest in mask use (low k_1, high k_2) (Figure S1). The model details are as follows:

$$\frac{dS^n}{dt} = -\frac{S^n}{N}\left(\beta_A(I_A^n + I_P^n + I_A^m r_t + I_P^m r_t) + \beta_S(I_S^n + I_S^m r_t)\right) + \mu S^m - \omega_A \frac{M}{N} S^n \tag{16}$$

$$\frac{dS^m}{dt} = -\frac{S^m r_s}{N}\left(\beta_A(I_A^n + I_P^n + I_A^m r_t + I_P^m r_t) + \beta_S(I_S^n + I_S^m r_t)\right) - \mu S^m + \omega_A \frac{M}{N} S^n \tag{17}$$

$$\frac{dE^n}{dt} = \frac{S^n}{N}\left(\beta_A(I_A^n + I_P^n + I_A^m r_t + I_P^m r_t) + \beta_S(I_S^n + I_S^m r_t)\right) + \mu E^m - \omega_A \frac{M}{N} E^n - \alpha_1 E^n \tag{18}$$

$$\frac{dE^m}{dt} = \frac{S^m r_s}{N}\left(\beta_A(I_A^n + I_P^n + I_A^m r_t + I_P^m r_t) + \beta_S(I_S^n + I_S^m r_t)\right) - \mu E^m + \omega_A \frac{M}{N} E^n - \alpha_1 E^m \tag{19}$$

$$\frac{dI_P^n}{dt} = \alpha_1 E^n + \mu I_P^m - \omega_A \frac{M}{N} I_P^n - \alpha_2 I_P^n \tag{29}$$

$$\frac{dI_P^m}{dt} = \alpha_1 E^m - \mu I_P^m + \omega_A \frac{M}{N} I_P^n - \alpha_2 I_P^m \tag{21}$$

$$\frac{dI_A^n}{dt} = \alpha_2 I_P^n P_a + \mu I_A^m - \omega_A \frac{M}{N} I_A^n - \gamma_A I_A^n \tag{22}$$

$$\frac{dI_A^m}{dt} = \alpha_2 I_P^m P_a - \mu I_A^m + \omega_A \frac{M}{N} I_A^n - \gamma_A I_A^m \tag{23}$$

$$\frac{dI_S^n}{dt} = \alpha_2 I_P^n (1 - P_a) + \mu I_S^m - \omega_S \frac{M}{N} I_S^n - \gamma_S I_S^n \tag{24}$$

$$\frac{dI_S^m}{dt} = \alpha_2 I_P^m (1 - P_a) - \mu I_S^m + \omega_S \frac{M}{N} I_S^n - \gamma_S I_S^m \tag{25}$$

$$\frac{dR^n}{dt} = \gamma_A I_A^n + \gamma_S I_S^n + \mu R^m - \omega_A \frac{M}{N} R^n \tag{26}$$

Supplementary Table 1. Parameter values used in the study

Parameter	Definition	Baseline value	References
R_0	Basic reproduction number for symptomatic individuals	2.5	[4,5,7,8]
$1/\alpha_1$	Time from exposed to pre-symptomatic	1	[4]
$1/\alpha_2$	Time from pre-symptomatic to symptomatic/ asymptomatic	5	[1-4]
P_a	The proportion of asymptomatic infections	30% (varied in simulations)	[9]
$1/\boldsymbol{\gamma}_A$	Time to recovery for asymptomatic infections	14	[4,10]
$1/\boldsymbol{\gamma}_S$	Time to recovery for symptomatic infections	14	[4,10,11]
r_t	Relative transmissibility with mask	Varied in simulations (=0.5 if not specified)	
r_s	Relative susceptibility with mask	Varied in simulations (=0.75 if not specified)	
Resource allocation model			
N_{70}	Proportion of people age 70+	7.6%	[12]
ϕ_e<br>ϕ_y	Death rate among symptomatics in 70+<br>Death rate among symptomatics in <70	9.7%<br>1.3%	[13]
δ	Detection rate of asymptomatic infections	44%	[14]
Supply & demand model			
B	Daily mask production	Varied in simulations	
$1/\mu$	Average time of wearing disposable mask	1	
d	The ratio of rate of wearing mask between symptomatic infected individuals and others (ω_S/ω_A)	Varied in simulations (=1000 if not specified)	

SUPPLEMENTARY FIGURES

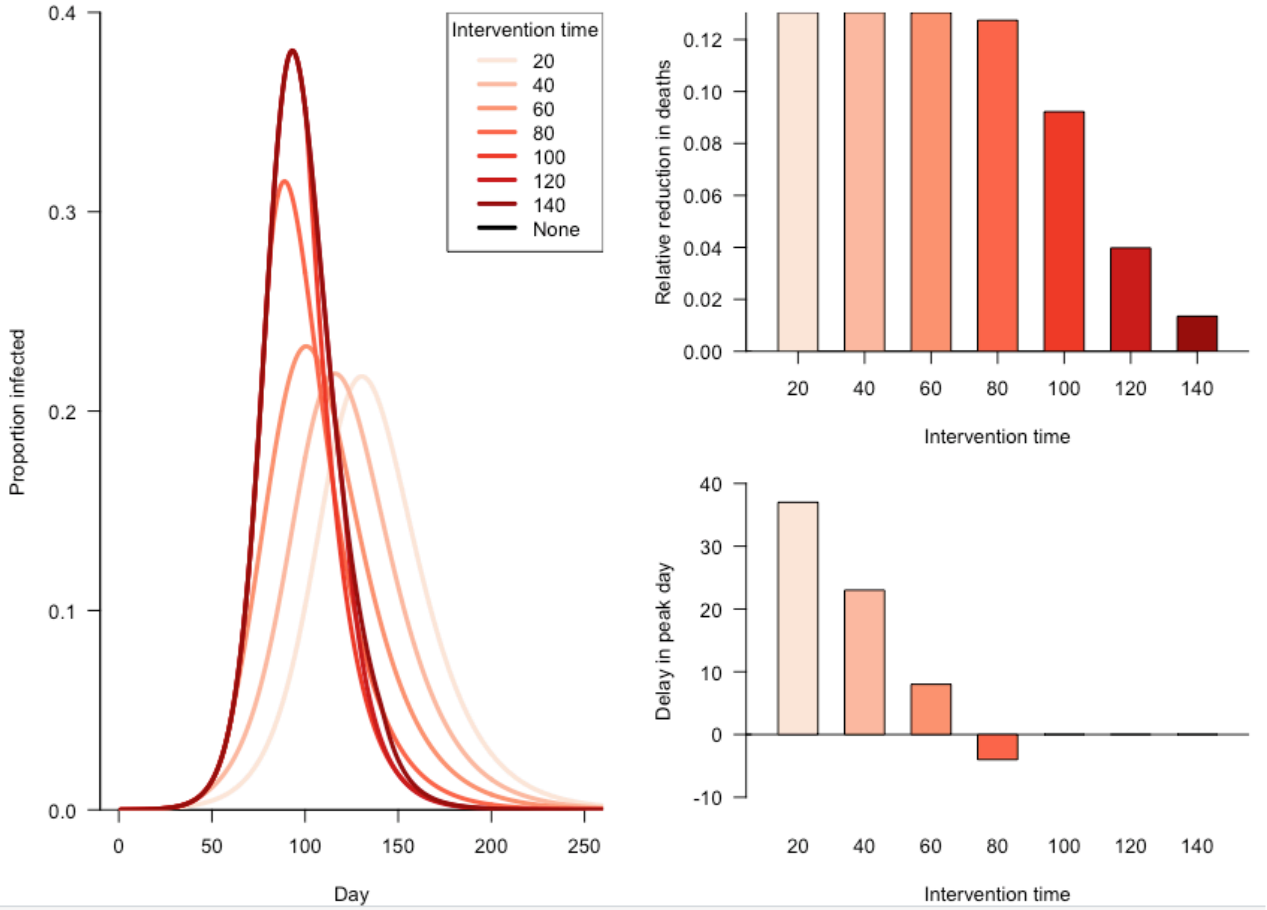

Supplementary Figure 1. Rapid introduction of face masks to the general population can reduce infections and delay the epidemic peak. For a range of intervention dates, 50% of the general population adopt face masks conferring 25% protection and 50% containment. Earlier interventions can reduce overall deaths, and delay the epidemic peak.

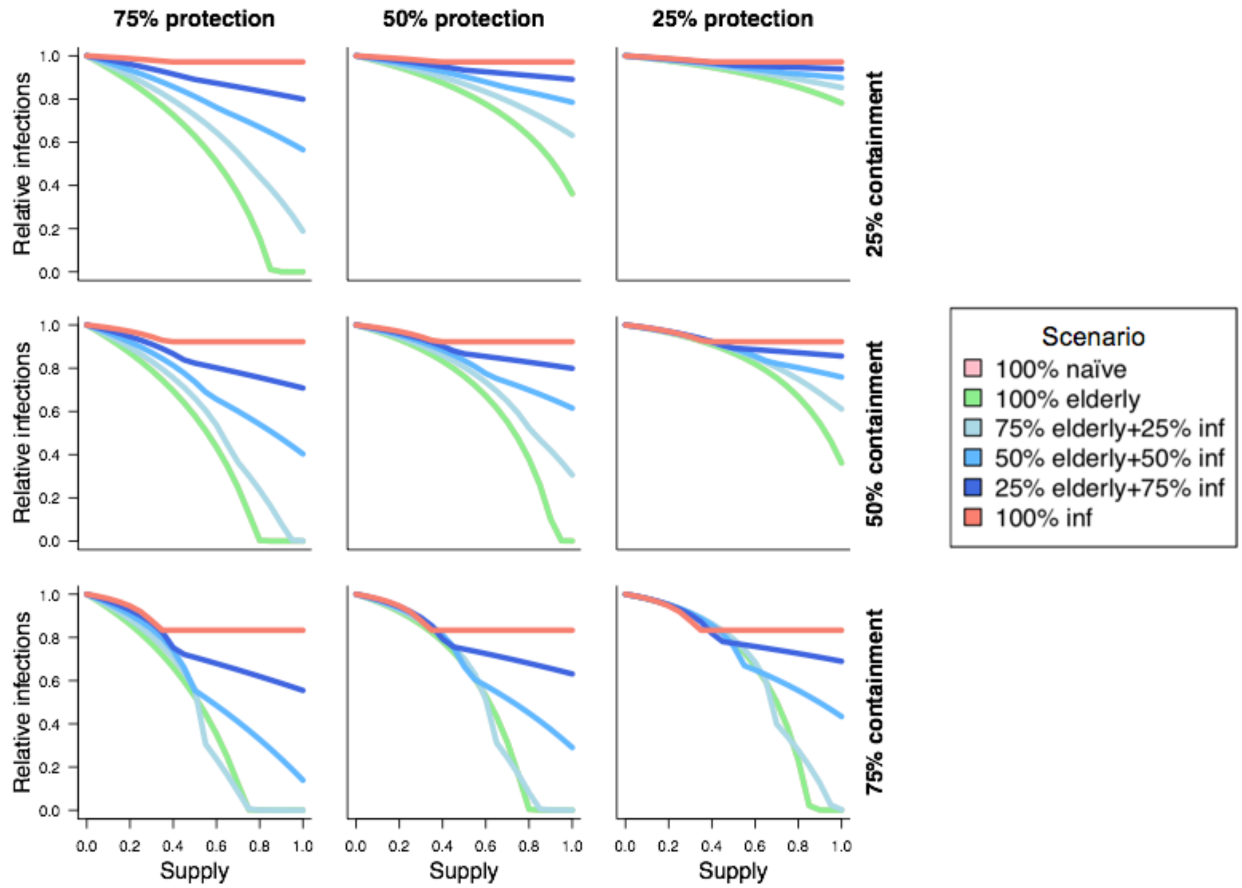

Supplementary Figure 2. Reduction in total infections under each of the described resource allocation strategy for a range of resource availability levels. Corresponds to Figure 2, showing here infections rather than deaths. Each panel represents intervention effectiveness in terms of relative susceptibility and transmissibility, with the bottom left panel denoting the most effective intervention (75% reduction in susceptibility and transmissibility) and the top right panel representing the least effective intervention (25% reduction in susceptibility and transmissibility). Resources are provided naïvely (pink), prioritized to the elderly (green), saved for detected cases (red), or balanced at different levels between healthy individuals, prioritizing the elderly, and detected cases (blue). 30% of infections are assumed to be undetected. See *Methods* for further details.

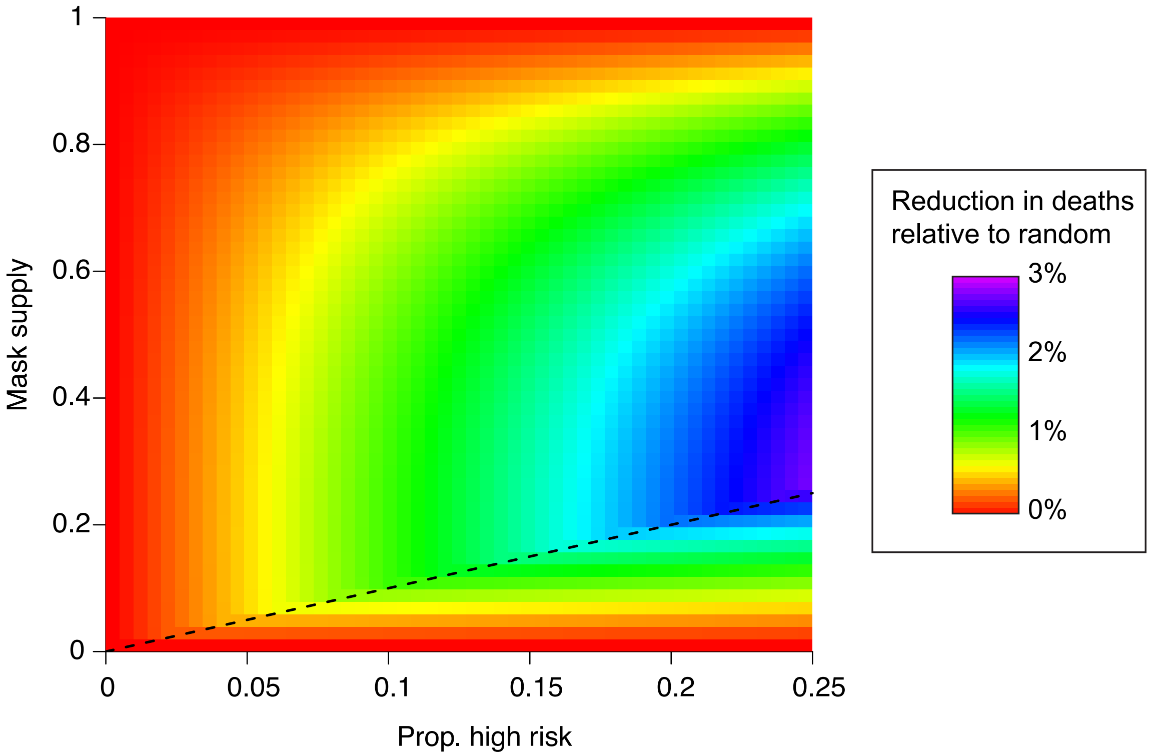

Supplementary Figure 3. Prioritized distribution to high risk individuals is increasingly optimal for larger high risk populations. For a mask providing 50% containment and 75% protection, we explored dynamics in populations with different 'high-risk' communities and mask supplies. For all scenarios considered, prioritized distribution to the elderly was the optimal strategy. This strategy was associated with greater reductions in deaths relative to the random distribution strategy, as shown by the colors. The dashed line indicates where mask supply is equal to the size of the high risk population.

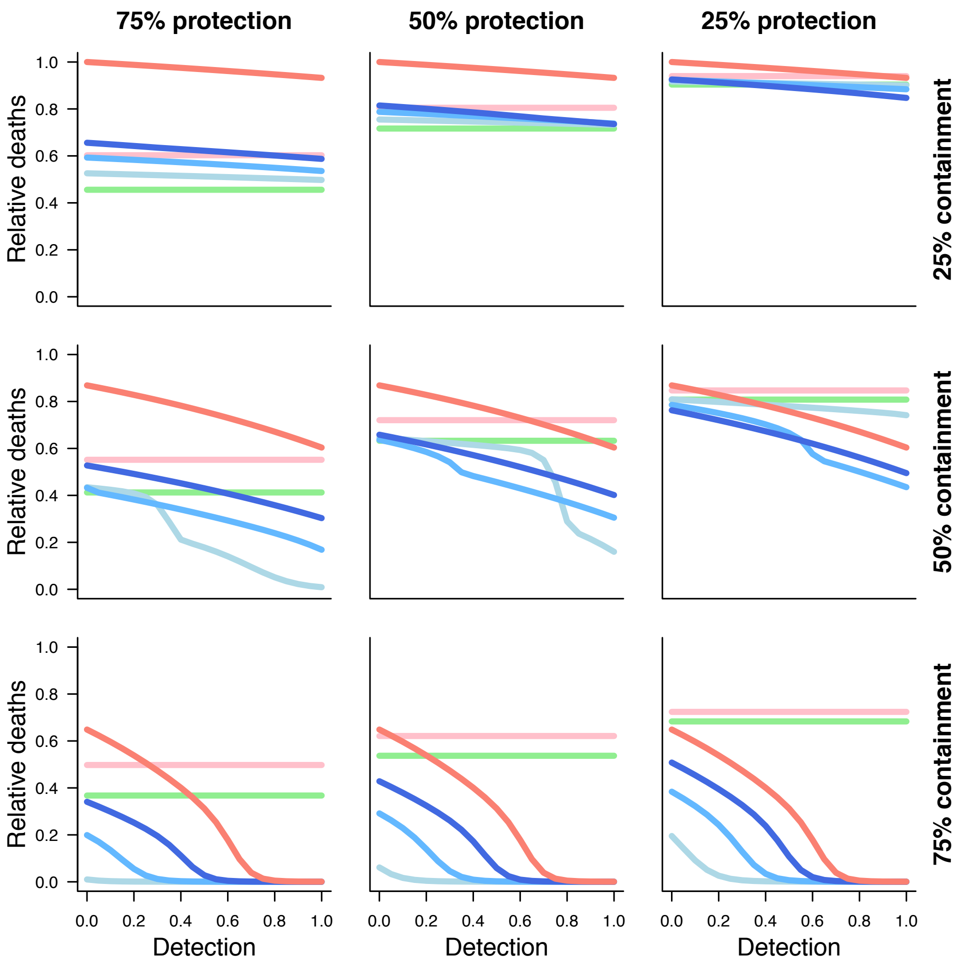

Supplementary Figure 4. Relative deaths decrease with a higher detection rate. For a range of mask effectiveness parameters, increased detection rates can improve outcomes under strategies in which infected persons are provided masks (strategy 4 [red] and strategies 3a-3c [blue]).

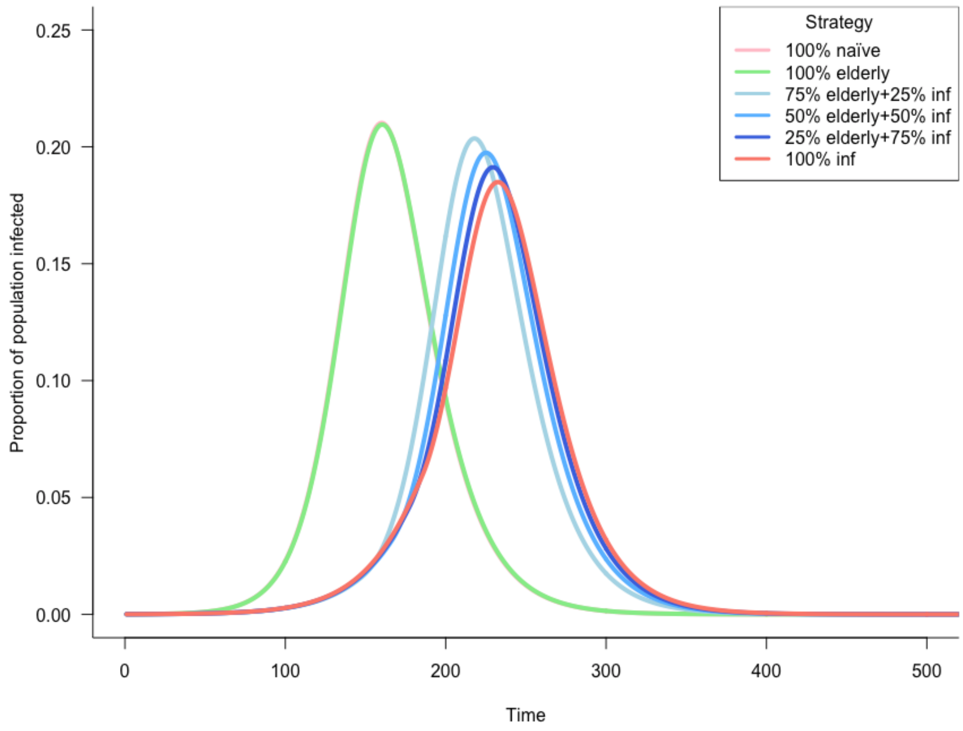

Supplementary Figure 5. Optimizing mask distribution strategy can delay epidemic peak. We demonstrate that, even low coverage (5% of the population) of a mask offering no protection and 40% containment, the epidemic peak can be delayed when retaining resources for detected cases.

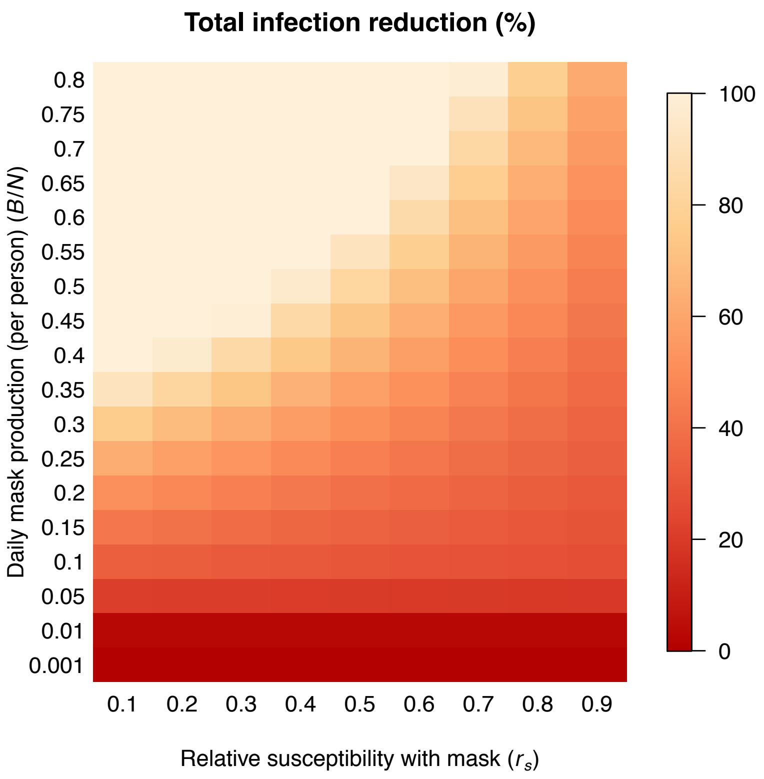

Supplementary Figure 6. Impact of mask production rates on final number of infections. The reduction in total numbers of infections for different levels of mask protection and mask production. (k_1, k_2) = (0.1, 100) here.

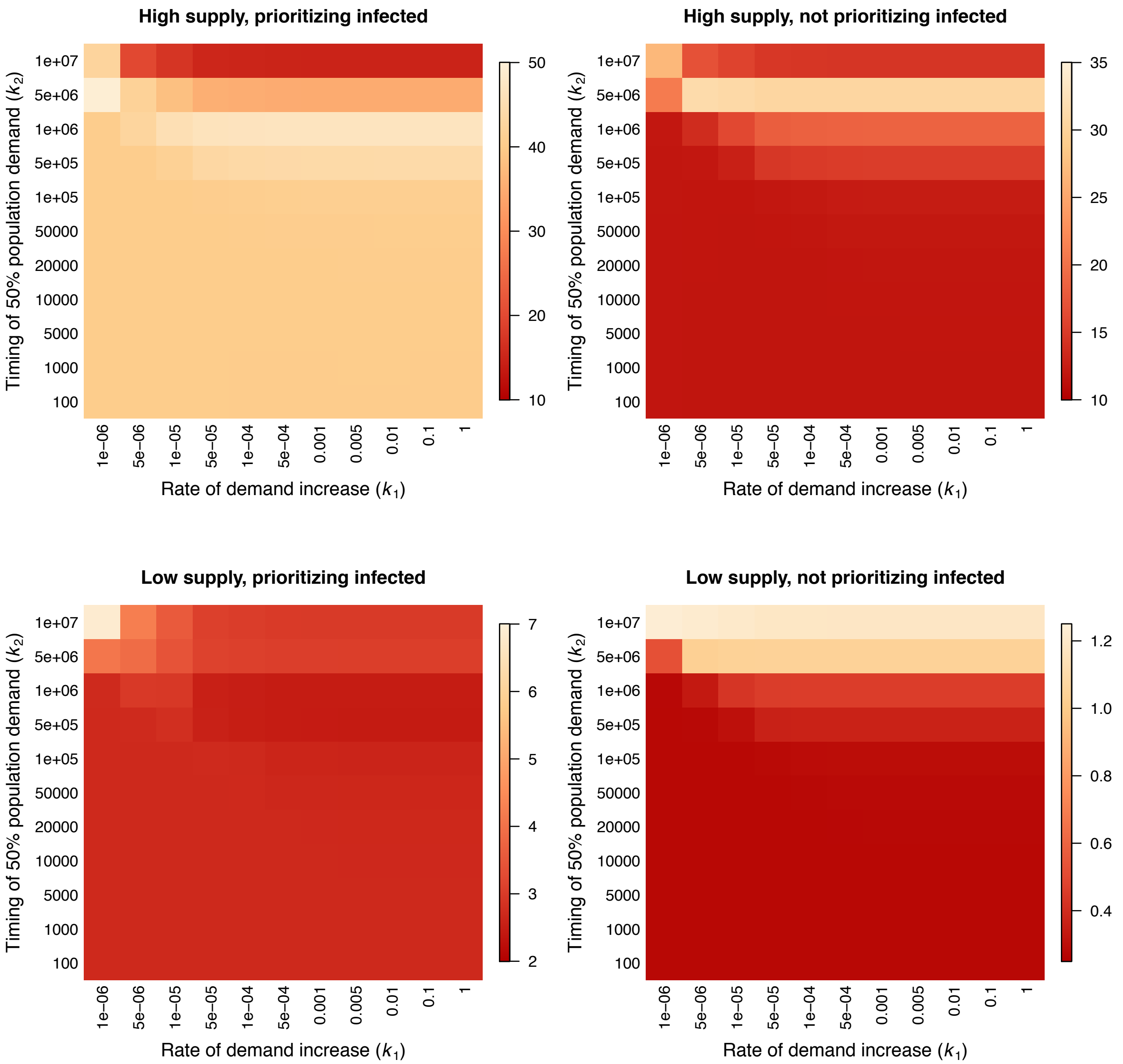

Supplementary Figure 7. Levels of reduction in total infection numbers compared to no mask condition under different demand functions. The level of total infection reduction (%) varies with the rate of demand increase (k_1) and the timing of 50% population demand on masks (k_2). Here shows total infection reduction under high (B/N= 30%) or low (B/N= 1%) mask production when prioritizing (left) or not prioritizing (right) masks for infectious cases. Generally, 'panic buying' is detrimental and prioritizing to infectious cases (setting $\omega_S > \omega_A$) is beneficial. N= 2.3×10^7 is used here.

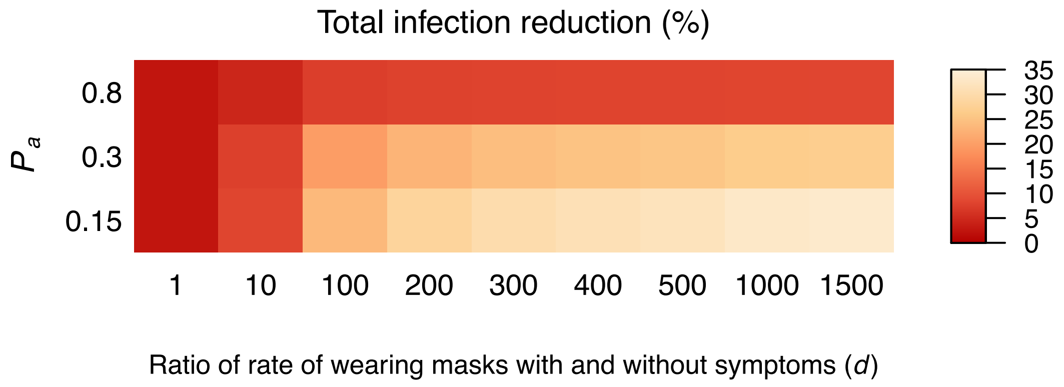

Supplementary Figure 8. The effect of prioritizing masks to infectious cases is less apparent if the proportion of asymptomatic infections is high. (k_1, k_2) = (0.1, 100) here.

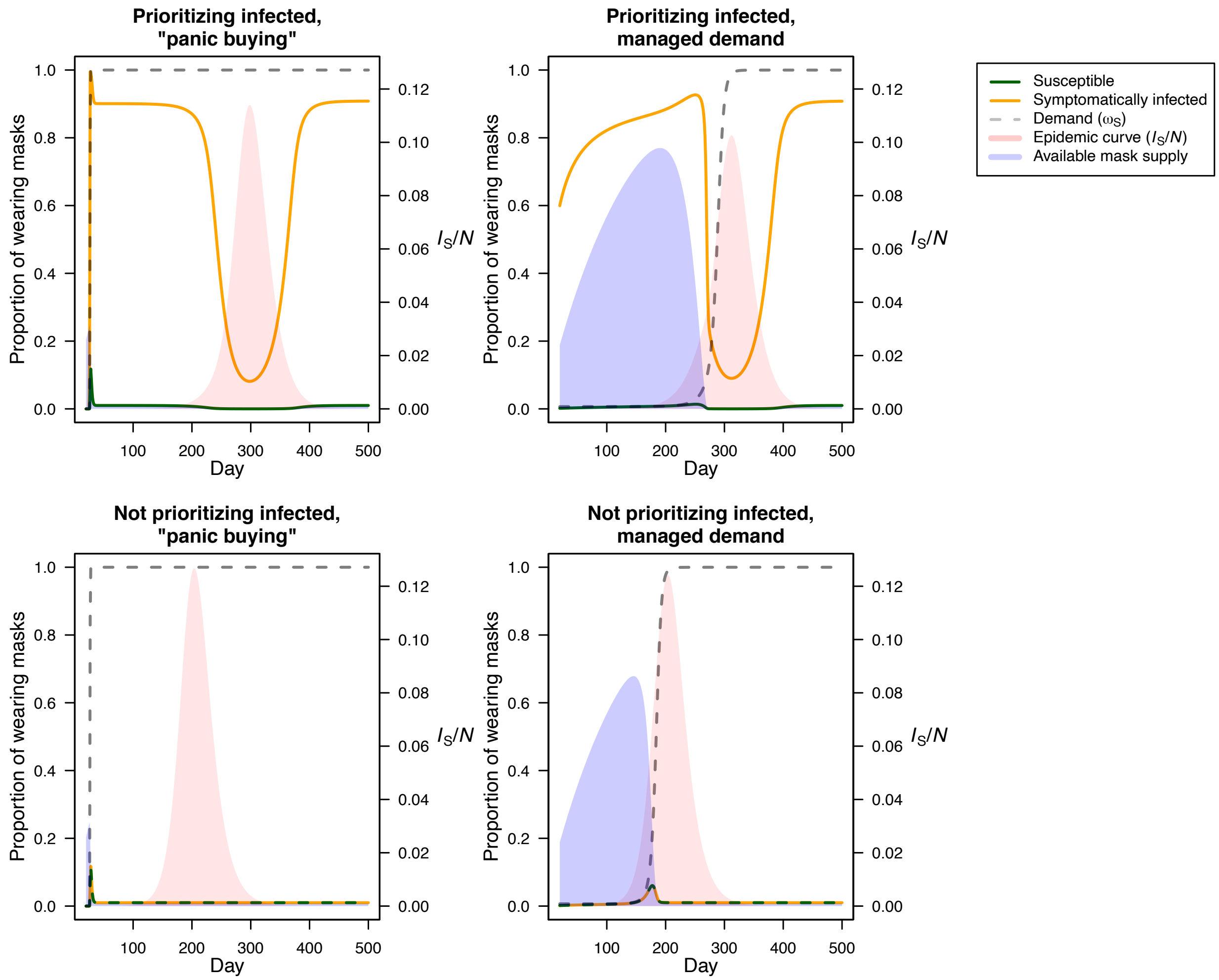

Supplementary Figure 9. Low mask production rate can limit the advantage of building up supplies. Epidemic curves (pink) under a 'panic buying' demand curve (left), and a more gradual managed demand curve (right) when prioritizing (top) and not prioritizing (bottom) masks for infectious cases. Demand is shown as a dashed grey line, while the relative available mask supply is shown in blue. The proportion of the susceptible and symptomatically infected persons wearing masks are shown as green and red lines, respectively. (k_1, k_2) are (1, 100) and (10^{-6}, 5×10^{6}) for "panic buying" and managed demand, respectively. While supplies are built up in the early phase of the epidemic (right), shortage still occurs during the outbreak if mask production rate is low (B/N = 1% here).

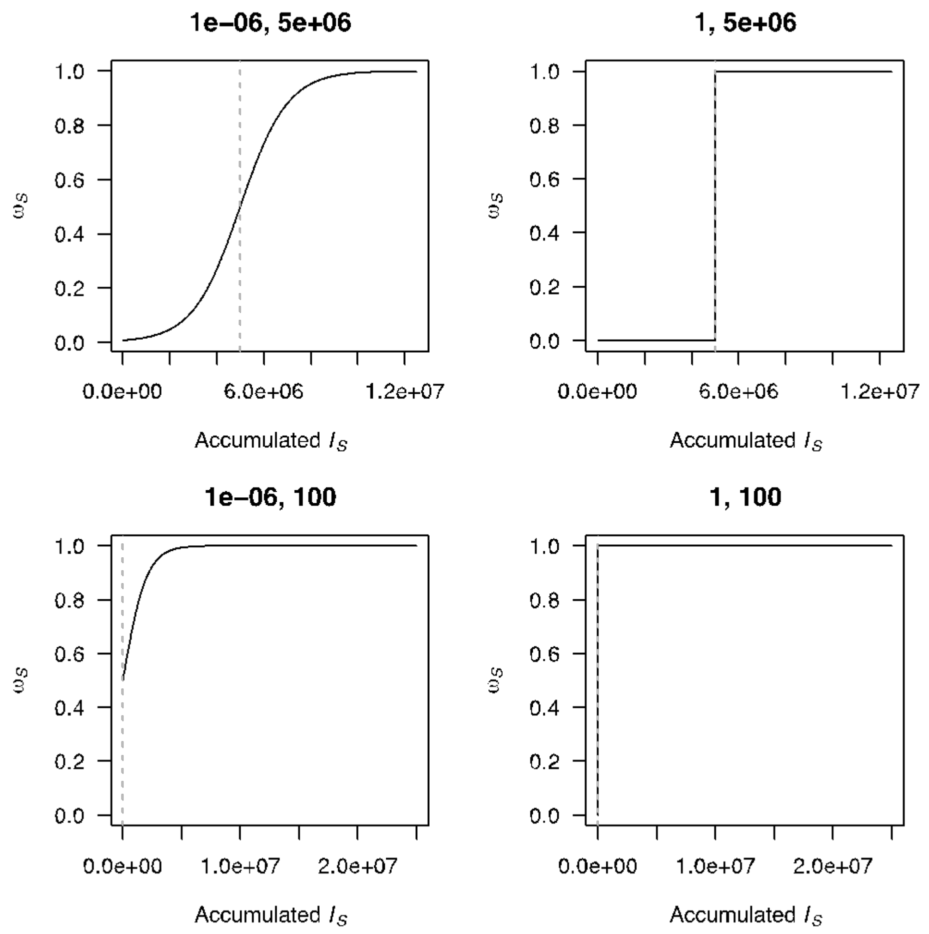

Supplementary Figure 10. Different parameterizations of demand dynamics. Different values of k_1 and k_2, provided as the title for each panel, result in different demand responses to increasing numbers of reported cases. A panic buying scenario can be approximated in the bottom right panel, where demand is maximal very early in the epidemic. A gradual increase in demand is shown in the top left panel, in which 50% of the population seek masks when the number of infections reaches 5 million.

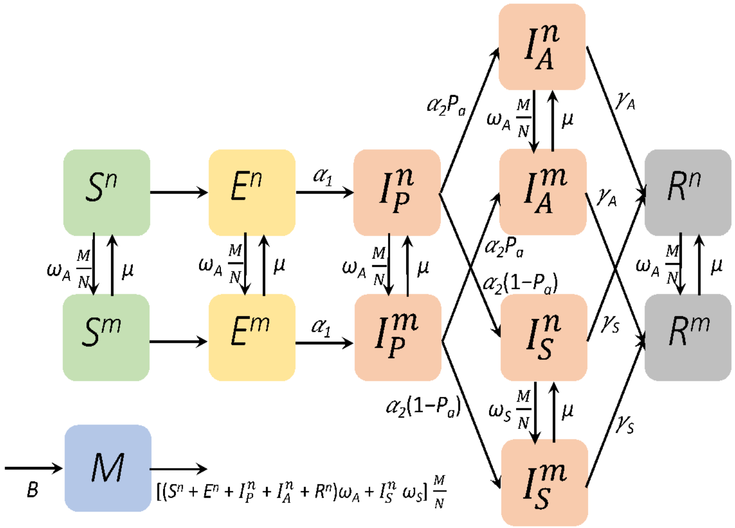

Supplementary Figure 11. Supply & demand model. Diagram of the supply & demand model showing the rates between each compartment.

Supplementary References

1 Bui, L. V. *et al.* Estimation of the incubation period of SARS-CoV-2 in Vietnam. *medRxiv*, 2020.2005.2009.20096800, doi:10.1101/2020.05.09.20096800 (2020).

2 Lauer, S. A. *et al.* The Incubation Period of Coronavirus Disease 2019 (COVID-19) From Publicly Reported Confirmed Cases: Estimation and Application. *Ann Intern Med* **172**, 577-582 (2020).

3 Zhang, J. *et al.* Evolving epidemiology and transmission dynamics of coronavirus disease 2019 outside Hubei province, China: a descriptive and modelling study. *Lancet Infect Dis* **20**, 793-802 (2020).

4 Anderson, R. M., Heesterbeek, H., Klinkenberg, D. & Hollingsworth, T. D. How will country-based mitigation measures influence the course of the COVID-19 epidemic? *Lancet* **395**, 931-934 (2020).

5 Mandl, M. M. K. Early Transmissibility Assessment of a Novel Coronavirus in Wuhan, China. *SSRN Electronic Journal*, doi:10.2139/ssrn.3524675 (2020).

6 Diekmann, O., Heesterbeek, J. A. & Roberts, M. G. The construction of next-generation matrices for compartmental epidemic models. *J R Soc Interface* **7**, 873-885 (2010).

7 European Centre for Disease Prevention and Control. *Novel coronavirus disease 2019 (COVID-19) pandemic: increased transmission in the EU/EEA and the UK – sixth update*, <https://www.ecdc.europa.eu/sites/default/files/documents/RRA-sixth-update-Outbreak-of-novel-coronavirus-disease-2019-COVID-19.pdf> (2020).

8 Chinese Center for Disease Control and Prevention. *Epidemic update and risk assessment of 2019 Novel Coronavirus 2020*, <http://www.chinacdc.cn/yyrdgz/202001/P020200128523354919292.pdf> (2020).

9 Nishiura, H. *et al.* Estimation of the asymptomatic ratio of novel coronavirus infections (COVID-19). *Int J Infect Dis* **94**, 154-155 (2020).

10 Zou, L. *et al.* SARS-CoV-2 Viral Load in Upper Respiratory Specimens of Infected Patients. *N Engl J Med* **382**, 1177-1179 (2020).

11 Beigel, J. H. *et al.* Remdesivir for the Treatment of Covid-19 — Preliminary Report. *New England Journal of Medicine*, doi:10.1056/NEJMoa2007764 (2020).

12 *The statistical data platforms of the three major censuses*, <census.dgbas.gov.tw> (2020).

13 Wu, Z. & McGoogan, J. M. Characteristics of and Important Lessons From the Coronavirus Disease 2019 (COVID-19) Outbreak in China: Summary of a Report of 72 314 Cases From the Chinese Center for Disease Control and Prevention. *JAMA* **323**, 1239-1242 (2020).

14 Omori, R., Mizumoto, K. & Nishiura, H. Ascertainment rate of novel coronavirus disease (COVID-19) in Japan. *Int J Infect Dis* **96**, 673-675 (2020).
